# Estimation of Potential HIV Transmission Risk in Recent Anal Intercourse Events among Men Who Have Sex with Men and Transgender Women in Bali, Indonesia

**DOI:** 10.3390/tropicalmed6030139

**Published:** 2021-07-22

**Authors:** Benjamin R. Bavinton, I Gusti Agung Agus Mahendra, John Kaldor, Matthew Law, Andrew E. Grulich, Pande Putu Januraga

**Affiliations:** 1 Kirby Institute, University of New South Wales, Sydney, NSW 2052, Australia; jkaldor@kirby.unsw.edu.au (J.K.); mlaw@kirby.unsw.edu.au (M.L.); agrulich@kirby.unsw.edu.au (A.E.G.); 2 Center for Public Health Innovation, Faculty of Medicine, Udayana University, Denpasar 80361, Bali, Indonesia; mhndrgst13@gmail.com (I.G.A.A.M.); januraga@unud.ac.id (P.P.J.); 3 Department of Public Health and Preventive Medicine, Faculty of Medicine, Udayana University, Denpasar 80361, Bali, Indonesia

**Keywords:** HIV, HIV prevention, men who have sex with men, transgender women, waria, Indonesia, sexual behaviour

## Abstract

In recent years, prevalence of Human Immunodeficiency Virus (HIV) has increased substantially in Bali, Indonesia, in men who have sex with men (MSM) and transgender women, known locally as ‘waria’. There are limited behavioural data in this population. We conducted a behavioural survey of MSM/waria in Bali in March–April 2018. Respondents were primarily recruited by HIV outreach workers. Respondents reported details about anal intercourse events with their last male/waria romantic partner and/or last male/waria casual partner (respondents could report on both if relevant). Statistical significance was tested with generalised estimating equations. Among 709 participants, median age was 27 (interquartile range = 24–31), and 92.1% were male and 7.9% were waria. One-third were born in Bali. Overall, 85.9% had ever had an HIV test; 55.1% reported being HIV-negative, 15.0% HIV-positive, and 30.0% had unknown serostatus. Most (86.5%) reported sex with men, 9.5% with waria, and 20.0% with women in the previous 6 months. Respondents described 703 anal intercourse events (397/306 with romantic/casual partners, respectively; 191 reported on both). Over half (56.5%) of the events were protected by condoms and 7.3% by biomedical prevention (2.6% by PrEP in either partner, 4.7% by HIV treatments in either partner). Thus, 36.3% of events involved unprotected condomless anal intercourse (40.8%/30.4% in romantic/casual partners, respectively). In multivariate analysis, unprotected condomless anal intercourse events were associated with romantic partners (*p* < 0.001), being born in Bali (*p* = 0.002), lower education (*p* = 0.013), believing that withdrawal before ejaculation is effective (*p* < 0.001), liking to use withdrawal (*p* = 0.021), and not liking condoms (*p* < 0.001). One-quarter of events had potentially reduced HIV transmission risk through non-condom-based risk reduction strategies, while 11.1% had no potential risk reduction. Events presenting the highest potential risk of HIV transmission were more commonly reported by respondents born in Bali.

## 1. Introduction

In 2018, UNAIDS reported that there were about 640,000 people living with HIV (PLHIV) in Indonesia [1]. In 2018–2019, integrated HIV bio-behavioural surveillance (IBBS) data indicated that the national HIV prevalence was 17.9% and 11.9% among men who have sex with men (MSM) and transgender women (known locally as ‘waria’), respectively [2]. In Denpasar, Bali, HIV prevalence is typically higher than the national average, with the 2019 IBBS reporting a prevalence among MSM of 38.1%, and the 2015 IBBS a prevalence of 34% among waria [2,3]. There are low rates of HIV testing, and in those diagnosed with HIV there are low rates of linkage to antiretroviral therapy (ART) and viral suppression. Nationally, it is estimated that only 51% of PLHIV have been diagnosed, and, of those, 17% are taking ART; the proportion with suppressed viral load is unknown but estimated to be only 1% [4]. However, HIV-positive MSM in Bali had better ART and viral suppression outcomes than other key populations and other locations in Indonesia [4]. For HIV-negative MSM/waria at high risk of infection, HIV pre-exposure prophylaxis (PrEP) is not available in Indonesia. Purchasing PrEP without a prescription is illegal and costly, and legally procuring PrEP pills from nearby countries such as Thailand is cost prohibitive for most potential PrEP users [5]. Previous work has demonstrated low awareness and use of PrEP among MSM in Bali, but relatively high interest in using it after being informed about it [5].

There are limited data available regarding sexual behaviour and HIV transmission risk among MSM and waria in Bali, or Indonesia more broadly [6]. Previous work has typically focused on condom use, and rarely examined use of biomedical prevention or non-condom-based HIV risk reduction strategies. Higher condom use has been associated with higher education, and sex with casual or commercial partners (as opposed to regular partners), while inconsistent condom use has been associated with drug use, depressive symptoms, and physical abuse as a child [7,8]. In a study using IBBS data, HIV infection was found to be associated with recent use of methamphetamines and infection with gonorrhoea or chlamydia [9]. Government-led IBBS surveys for key populations are conducted every two years in only three to six districts nationally, and locations alternate each round, making it difficult to obtain timely trend data. IBBS surveys include only about 200 to 250 members of each key population in each location and include minimal questions about sexual behaviour. While a valuable tool for gaining a repeated snapshot of the Indonesian epidemic in key populations, the IBBS is limited in its ability to provide timely, detailed, MSM- and waria-specific information required by local community organisations, non-government organisations, governments, and health services/clinics. Thus, there is an urgent need for detailed, high quality behavioural data regarding MSM and waria in Indonesia. Additionally, there is a need for data specific to the Bali setting given the important differences between Bali and other Indonesian provinces, such as the difference in dominant religion (Hinduism rather than Islam) and that Bali is a major international tourist destination.

In this analysis, we aimed to describe the characteristics of MSM/waria living in Bali, Indonesia, to examine the level of potential HIV transmission risk at the most recent anal intercourse events with romantic and casual partners, and to determine factors associated with anal intercourse events unprotected by either condoms or biomedical HIV prevention (i.e., ART or PrEP).

## 2. Materials and Methods

The Survei Kesehatan Seksual Indonesia (SeKSI) Study was conducted in March and April 2018 in Bali province, Indonesia. Respondents were primarily recruited through online and face-to-face outreach, and ‘snowball’ sampling (peer referral). Recruitment was conducted by 32 trained outreach workers from five organisations: Yayasan Kerti Praja (non-government HIV/STI clinic), Yayasan Gaya Dewata (gay community organisation), Bali Medika Clinic (private GP clinic), and Yayasan Bali Peduli (non-government HIV/STI clinic). The online survey instrument was hosted by SurveyGizmo, and was available in Bahasa Indonesia and English. Respondents could complete the survey on their own devices (either in the field or at home) or on the outreach workers’ devices. Respondents were reassured that outreach workers could not access their responses. Respondents provided informed consent at the start of the survey. At the end of the survey, they were invited to provide their mobile phone number to receive IDR 25,000 in mobile phone credit for their participation; this was completely optional. Ethical approval was provided by the Human Research Ethics Committee of the University of New South Wales, Sydney, Australia, and the Ethics Committee of the Faculty of Medicine, Udayana University, Bali, Indonesia.

To be eligible for participation, respondents had to be a cisgender male who self-identified as ‘gay’ or ‘bisexual’, be a waria/transgender woman, or be a cisgender male who had had sex with a male or waria/transgender woman partner in the previous 12 months. They also had to be at least 18 years of age, living in Bali province or have a plan to stay for at least 6 months, and able to complete the survey in Bahasa Indonesia or English.

The survey included items on: demographics; strength of identification with sexual identities (on a scale from 0 = not at all to 3 = very much); gender identity; HIV testing; ART, viral load and CD4 count in HIV-positive respondents; PrEP use in HIV-negative respondents; sex work in the previous 6 months (including sex in exchange for money or gifts, both incidental and as the main source of income); sexual behaviour in the last 6 months (sex with men, women, and/or waria); where respondents met male/waria sex partners in the last 6 months (mobile apps, social media, gay bar/club, other bar/club, gay sauna, and/or a public area); alcohol use, recreational drug use (including ecstasy, cannabis, crystal methamphetamine, cocaine, and/or heroin), and any drug use for the purposes of sex in the last 6 months; attitudinal items (belief in effectiveness of withdrawal before ejaculation to prevent HIV; how much the respondent likes using withdrawal and condoms); and a two-item scale on social engagement with MSM/waria (number of MSM/waria friends and amount of free time spent with them) [5], adapted from a reliable scale used in HIV research with gay and bisexual men [10].

Respondents who reported having a romantic partner in the last 6 months, and respondents reporting sex with any casual partners in the last 6 months, were asked about their last anal intercourse event with a romantic or casual partner separately. Respondents could complete these items on both partner types. Items included: partner demographics; partner serostatus; partner ART or PrEP use, for HIV-positive and HIV-negative partners, respectively; and sexual behaviour at the event (including condom use, sexual positioning, withdrawal, ejaculation, and group sex). Each anal intercourse event was then classified into mutually exclusive categories: condom use; condomless anal intercourse (CLAI) protected by ART or PrEP in either partner or both partners; and CLAI unprotected by ART or PrEP in both partners (termed ‘unprotected’ CLAI events, representing a potential HIV transmission risk). Within this last category, events were further classified into events with any potential non-condom-based risk reduction (including strategic positioning, having concordant HIV status, and withdrawal before ejaculation) or CLAI events with no potential risk reduction (also termed ‘highest-risk CLAI’).

In the analysis, we first described the characteristics of the sample overall, and stratified by place of birth (Bali or elsewhere in Indonesia); chi-square, Kruskal–Wallis, or t-tests were used for these comparisons. All anal intercourse events were classified by the schema described above: for all events combined, by partner type (romantic versus casual), and by respondent place of birth (Bali or elsewhere); these comparisons used chi-square tests. We then determined factors associated with protected events (protected by condoms or protected by ART or PrEP in either partner) and unprotected events (events involving CLAI not protected by ART or PrEP in both partners) using generalised estimating equations (GEE) to control for inter- and intra-subject variability. Events with both romantic and casual partners were included in this model; individual respondents could have none, one or two events in this model. We report odds ratios (OR) for bivariate associations, adjusted ORs (aOR) for multivariate associations, 95% confidence intervals (CI), and *p*-values. Associations with *p*-values less than 0.1 in bivariate models were block entered into the multivariate model. Type I error was *p* < 0.05 for all statistical tests. Analysis was performed using Stata (version 14.2, StataCorp, College Station, TX, USA).

## 3. Results

In total, 1076 surveys were opened or commenced. However, 248 surveys were duplicates (due to a technical issue) or repeated surveys, and 119 were excluded (10 were from cisgender female respondents; 93 had no data at all; 8 were from respondents born outside of Indonesia; and 8 had no country of birth information). This resulted in a final sample of 709 respondents. The characteristics of the sample, along with a comparison of those born in and outside of Bali, are presented in Table 1. Median age was 27 (IQR = 24–31). Most (92.1%) respondents were male, with 7.9% identifying as waria, transgender or female. Those born in Bali were more likely to identify as waria (*p* = 0.012). Among the male respondents, those born in Bali were less likely to identify as ‘gay’ (*p* = 0.006) and more likely to identify as ‘heterosexual’ (*p* < 0.001). One-third were born in Bali, 40.9% were born in Java, and the remaining quarter were born elsewhere in Indonesia. About one-quarter of the respondents had completed a university degree and this was higher among the Bali-born (*p* = 0.047). Most (86.7%) respondents were working, with a higher proportion among the non-Bali-born respondents (90.0% versus 80.3%, *p* < 0.001). Overall, 29.2% reported a monthly income of < 2 million Indonesian rupiah (IDR; approximately USD $140), 31.0% of 2–3 million IDR, 30.8% of 3–5 million IDR, and 9.0% of 5+ million IDR; the Bali-born were more likely to earn < 2 million IDR (*p* = 0.011). In the previous 6 months, 16.9% had done some incidental sex work, while 11.1% reported sex work as their main source of income. 

Over 90% of the respondents were recruited to the study via HIV outreach workers, and 89.4% and 77.7% had had contact with these workers face-to-face or online, respectively. Overall, 14.1% had never been tested for HIV, and this was higher (20.5%) among the Bali-born than the non-Bali-born (10.9%, *p* = 0.001). Most (71.5%) reported that they had been tested in the previous 12 months, while 14.4% reported their last test was over 12 months ago. At their last test, 15.0% were HIV-positive, 55.2% were HIV-negative, 15.0% were untested or did not collect their test result (with 14.0% untested and 1.0% who did not collect the result), and 15.0% did not report their HIV status. Among the 106 HIV-positive respondents, 93.4% were taking ART. Half had never had a viral load test or did not know the result of the last test, one-third (32.1%) had undetectable viral load at their most recent test (or 64.2% of those who received a result), and 17.9% had detectable viral load (or 35.9% of those who received a result). CD4 count testing was more common, with just 6.6% of HIV-positive respondents never having had a test or not knowing the result; 49.1% had a CD4 count < 350 and 44.3% had a CD4 count of 350 or more. Of the 391 HIV-negative respondents, 10 (2.6%) were taking PrEP. 

Most (86.5%) reported sex with male partners in the previous 6 months, while 9.5% reported sex with waria partners, and 20.0% reported sex with women. Sex with waria and with women were more common among Bali-born respondents (*p* < 0.001, respectively). The most common method of meeting male/waria sexual partners in the previous 6 months was on gay mobile applications (58.4%), followed by social media (56.6%), gay bars/clubs (31.7%), public areas (26.5%), other bars/clubs (24.7%), and gay saunas (20.2%). There were differences between the Bali-born and non-Bali-born: Bali-born respondents were more likely to use social media (*p* = 0.027) and public areas (*p* = 0.005), and less likely to use gay mobile applications (*p* = 0.019), gay bars/clubs (*p* = 0.007), and gay saunas (*p* = 0.001). In the previous 6 months, 41.9% reported using alcohol, 14.5% reported using any recreational drugs, and 9.2% any drugs for the purposes of sex. 

**Figure 1:**
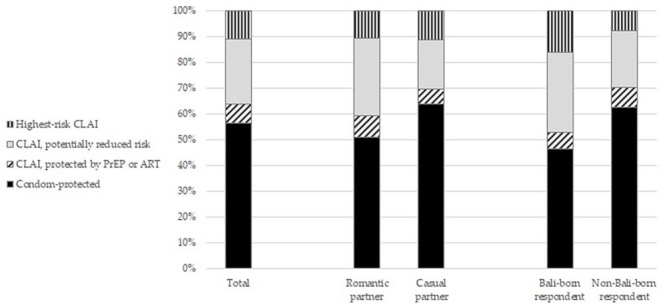
Comparison of classifications of the most recent anal intercourse event: total (*n* = 703); by partner type (romantic partner, *n* = 397; casual partner, *n* = 306); and by respondent place of birth (Bali-born, *n* = 263; non-Bali-born, *n* = 440). CLAI, condomless anal intercourse; PrEP, pre-exposure prophylaxis; ART, antiretroviral therapy.

In the previous 6 months, 411 respondents (58.0%) reported having had any sex with a romantic partner, and of these, 397 (96.6%) reported on their last anal intercourse event with a romantic partner; while 327 respondents (46.1%) reported having had any sex with a casual partner, and of these, 306 (93.6%) reported details of their last casual anal intercourse event. Overall, 191 respondents reported on separate recent anal intercourse events with both types of partner. Respondents reported on a total of 703 anal intercourse events. Of these events, respondents reported that they used condoms at 397 of them (56.5%), and a further 51 events (7.3%) were protected by at least one of the partners being on ART or PrEP (Table 2; Figure 1). Thus, 448 events (63.8%) were considered to be ‘protected’. There were 255 events (36.3%) involving CLAI unprotected by ART or PrEP (hereafter referred to as ‘unprotected CLAI events’). However, within these 255 unprotected CLAI events, 177 (69.4% of unprotected CLAI events, or 25.2% of all events) were when there was some form of HIV risk reduction such as strategic positioning (used in 40 events), concordant HIV status (also known as ‘serosorting’, used in 99 events), or withdrawal before ejaculation (used in 86 events). Use of these risk reduction strategies was not mutually exclusive. Thus, there were 78 events (30.6% of unprotected CLAI events, or 10.8% of all events) that involved CLAI with no potential risk reduction; these events could be considered as having the highest potential risk for HIV transmission. In bivariate GEE models, unprotected CLAI events were more likely in those: born in Bali; with a monthly income of <2 million IDR; who had engaged in any sex work in the previous 6 months; who reported sex with women and/waria in the previous 6 months; who had infrequent contact with HIV outreach workers in the previous 12 months; who believed withdrawal is an effective HIV prevention method; and those who liked using withdrawal for HIV prevention (Table 3). Unprotected CLAI events were also more likely when with romantic partners. On the other hand, unprotected CLAI events were less likely in those: with a university education or diploma; with lower social engagement with other MSM/waria; and who liked using condoms. Factors independently associated in the multivariate GEE model were: being born in Bali; having less than a university education or a diploma; disliking condoms; belief that withdrawal is effective; liking to use withdrawal; and the event being with a romantic partner (Table 3).

Given that two of the covariates with the strongest associations with unprotected CLAI events were partner type (romantic versus casual) and birthplace of the respondent (Bali versus outside of Bali), we compared the classifications of the anal intercourse events in more detail (Table 2; Figure 1).

First, at the last anal intercourse event, condoms were used at 50.9% of events with a romantic partner and 8.3% involved CLAI that was protected by either ART or PrEP. By contrast, condoms were used at 63.7% of anal intercourse events with casual partners (*p* = 0.001 for the comparison with romantic partners), and 5.9% of events involved CLAI but were protected by either ART or PrEP. The remaining 40.8% of events with romantic partners involved CLAI unprotected by ART or PrEP. However, within these 162 unprotected CLAI events, 119 (73.5% of unprotected CLAI events, or 30.0% of all events) were when there was some form of HIV risk reduction. For events with casual partners, 30.4% (*n* = 93) were unprotected CLAI events (*p* = 0.004 for the comparison with romantic partners). Of these, 58 (62.4% of unprotected CLAI events, 19.0% of all events) involved some form of risk reduction. In events with romantic partners and casual partners respectively, there were 43 events (26.5% of unprotected CLAI events, or 10.8% of all events) and 35 events (37.6% of unprotected CLAI events, 11.4% of all events) that involved CLAI with the highest potential risk for HIV transmission and with no potential risk reduction (*p* = 0.800).

Second, at the last anal intercourse event, 122 (46.4%) Bali-born respondents used condoms compared to 275 (62.5%) non-Bali-born respondents (*p* < 0.001), and 17 (6.5%) Bali-born respondents had CLAI protected by ART or PrEP compared to 34 (7.7%) non-Bali-born respondents (*p* = 0.045). Thus, a higher proportion of events in Bali-born respondents (*n* = 124, 47.2%) involved CLAI not protected by ART or PrEP compared to non-Bali-born respondents (*n* = 131, 29.8%; *p* < 0.001). In these unprotected CLAI events, 81 (65.3% of unprotected CLAI events, or 30.8% of all events) involved some form of behavioural risk reduction in the Bali-born, compared to 96 (73.2% of unprotected CLAI events, or 21.8% of all events) among the non-Bali-born (*p* = 0.168). Use of withdrawal was higher in the Bali-born (*p* = 0.019). Bali-born respondents had a higher proportion of the highest risk unprotected events with no potential risk reduction (*n* = 43, 16.3%) than the non-Bali-born (*n* = 35, 8.0%, *p* < 0.001).

## 4. Discussion

We found that in recent anal intercourse events among MSM and waria in Bali, Indonesia, over one-third of events were unprotected by condoms or biomedical prevention, and just over one-tenth of events represented the highest potential HIV transmission risk with no potential risk reduction. We found that the proportion of unprotected CLAI events was higher at events with romantic partners compared to with casual partners, and among respondents born in Bali compared to those born elsewhere in Indonesia. We also identified several factors associated with reporting more unprotected CLAI events, including lower education, having less positive attitudes towards condoms, and having more interest in withdrawal. As one of the largest samples of MSM and waria in Bali to date, our study provides insights into the characteristics of these populations, including important differences between those born in Bali and those born elsewhere in Indonesia. 

With casual partners, we found that over 60% of recent anal intercourse events were protected by condoms, similar to what has previously been reported in other Indonesian studies [7,9]. Use of condoms was lower with romantic partners, in about 50% of events. This pattern, whereby condoms are used less often in romantic relationships, has been commonly reported in research with MSM internationally [11,12,13]. Greater familiarity with romantic partners, as well as the desire for increased intimacy, may be key motivators [14]. Often, CLAI in the context of primary romantic relationships can be safe with respect to HIV transmission, such as when using a non-condom-based risk reduction strategy such as ‘negotiated safety’ in HIV-negative seroconcordant relationships [15], or relying on an undetectable viral load in serodiscordant relationships [16]. However, it is important to note that HIV infections in the context of relationships may still be prevalent in many countries, especially in settings where non-condom-based risk reduction strategies have not been openly promoted, as is the case in Bali where HIV prevention education has essentially focused only on condoms. HIV education efforts may need to recognise the perceived differences between casual and romantic sexual contexts. 

Respondents who were born in Bali used condoms at less than half of recent anal intercourse events, compared to over 60% in those born elsewhere in Indonesia. This meant that nearly half of the events were unprotected by condoms or biomedical prevention among the Bali-born respondents, while less than a third were unprotected in the non-Bali-born. The proportion of events classified as having the highest potential transmission risk was also higher in the Bali-born (16%) than the non-Bali-born (8%). This difference in potential transmission risk occurred even though contact with HIV outreach workers was similar in the two groups. While it is not yet entirely clear why the Bali-born respondents reported these higher levels of potential transmission risk, we found some important differences between the Bali-born and non-Bali-born that may be implicated. In our sample, Bali-born respondents were more likely to be waria, and also more likely to report sex with women and with waria. The male respondents were less likely to identify with the term ‘gay’, and more likely to identify with the term ‘heterosexual’. We also observed differences in where they found sex partners: the Bali-born respondents tended to use ‘gay community’ approaches less often (such as gay mobile apps, gay bars/clubs, and gay saunas) and use social media and public places more often. Taken together, this suggests that there may be important differences between those who were born and grew up in Bali compared to those who were born elsewhere in Indonesia. Of the cities in Indonesia, Bali is often seen as a place that is more permissive of gay life than other parts of Indonesia. Many people from all over Indonesia migrate to Bali to work in the tourist industry, meaning that they are away from family and the friends they grew up with. For many MSM and waria, this may be motivated in part by the desire to establish a new, somewhat more open life in Bali [17]. For Bali-born respondents, this freedom may be more difficult to access, due to close proximity to family and childhood friends, community responsibilities, and pressures to marry. Different approaches are likely needed for the various kinds of MSM/waria living in Bali: those living a relatively open life in Bali are very different from those who are closeted or married; those born in Bali appear more likely to be in this second group. 

We found some associations with potential HIV transmission risk which supported previous work among MSM and waria in Indonesia, including that higher education was associated with decreased transmission risk [7,9]. In bivariate analysis, we also found that lower income was associated with higher potential transmission risk. As in other studies, our data showed that participation in sex work and sex with women or waria were predictive of higher risk [7,9]. By contrast to these studies, drug use (including drug use for the purposes of sex) was not associated with higher potential transmission risk in our data [7,8,9,18]. Similarly, while group sex has been a predictor of higher risk in MSM [19], whether or not anal intercourse events involved group sex was not associated with the event being unprotected by condoms or biomedical prevention in our data. We found that other statistically significant predictors of potential HIV transmission risk in our study included believing withdrawal is an effective HIV prevention method and liking to use withdrawal, and that liking to use condoms was predictive of lower potential transmission risk. HIV outreach workers in Bali may need to actively counter beliefs about the effectiveness of withdrawal as a means of HIV prevention [15]. Finally, in bivariate analysis, the degree of social connection respondents had to other MSM/waria was associated with lower potential HIV transmission risk. Higher social engagement has been associated with condom use, HIV testing, and PrEP in international research [20,21,22], as well as with greater interest in using PrEP among MSM/waria in Bali [5]. 

HIV treatment uptake among the HIV-positive respondents was high (93%), supporting national work which has demonstrated highest treatment uptake among MSM in Bali compared to other key populations and other settings in Indonesia [4]. The level of HIV testing observed in our data was also high in comparison to other studies of Indonesian MSM/waria [9,23], especially among MSM [23]. These proportions may have been impacted by the method of recruiting respondents via HIV outreach workers. By contrast, since PrEP is not available in Indonesia, it is unsurprising that we observed a very low proportion of HIV-negative respondents reporting PrEP use, at less than 3%. We also found that only 2.6% of anal intercourse events were protected by PrEP in either the respondent or their partner. This supports similar findings of very low PrEP use among MSM/waria in Bali [5], and Indonesia more broadly [24]. Due to this lower use of PrEP, CLAI protected by biomedical prevention was still a minority practice in our study. This is in stark contrast to some other settings with high PrEP uptake, where biomedical prevention has now overtaken consistent condom use as the most commonly used prevention method for casual sex encounters [25]. However, it is important to also note that among those who use it, reliance on biomedical prevention may not be as trustworthy in Bali as in some other settings internationally. The available HIV cascade data from Indonesia suggests that ongoing adherence to ART among HIV-positive individuals is not optimal [4], while for PrEP users, difficulties in accessing PrEP may lead to poorer adherence or breaks in use. Furthermore, as mentioned above, HIV education in Bali has focused almost exclusively on condoms, which may mean, for example, that the preventive benefits of ART may not be widely known. An urgent priority for HIV education in Bali is the wider dissemination of information about these benefits, which may assist in motivating ART uptake and adherence. Greater access to PrEP is also critical [26].

Our findings should be considered in light of some limitations. As a convenience sample, the findings are not necessarily representative of all MSM and waria living in Bali. In particular, 90% of respondents were recruited by HIV outreach workers, which means they may have had higher levels of HIV testing, ART, and knowledge about HIV prevention than in the general MSM/waria population. Additionally, the low number of waria in the study meant that we could not meaningfully disaggregate the waria respondents from the MSM. As with all self-report surveys, social desirability may have impacted some responses, although this was mitigated by the fact it was an anonymous self-completed survey rather than an interview. Respondents were reassured that their answers could not be seen by the outreach workers recruiting them. For some of the items, respondents may have had difficulties recalling the precise details of the anal intercourse encounters. For the use of non-condom-based risk reduction strategies, we had limited information about how intentional the use of these strategies was. The survey asked about the last occasion of sex with romantic and casual partners separately, but did not ask about the last occasion with sex workers or sex work clients; thus, we were unable to explore the potential HIV transmission risk in such transactional sex encounters. The study data were collected in 2018 and since then, PrEP use has grown rapidly among MSM and transgender women globally [26]. PrEP has had major impacts on sexual cultures, including changes in condom use, among MSM [25]. However, as PrEP is not widely available in Indonesia [5,26], usage is very low, and PrEP is unlikely to have yet had a large impact on sexual behaviour in Bali. Finally, as HIV testing was not conducted as part of this study, there was a relatively large proportion of participants with unknown HIV status.

## 5. Conclusions

Our analysis indicated that over one-third of recent anal intercourse events among MSM and waria in Bali, Indonesia, represented a potential risk for HIV transmission, and that this risk was greater in events with romantic partners and among respondents who were born in Bali. We found that PrEP use was minimal. HIV education efforts should incorporate more information about biomedical HIV prevention. Given that non-condom-based risk reduction strategies such as having the same HIV status and withdrawing before ejaculation were common among those having condomless sex, HIV education should also openly discuss the relative risks and merits of these strategies with MSM and waria. However, ultimately, PrEP scale-up is urgently needed to reduce HIV transmission in these populations.

## Figures and Tables

**Table 1:** Sample characteristics for the total sample and stratified by place of birth.

	Total Sample (*n* = 709)	Born in Bali (*n* = 239)	Born Outside Bali (*n* = 470)	*p*-Value
Location of birth				NA
Bali	239 (33.7)	239 (100.0)	0 (0.0%)	
Java	290 (40.9)	0 (0.0%)	290 (61.7)	
Other	180 (25.4)	0 (0.0%)	180 (38.3)	
Age—median (IQR)	27 (24–31)	26 (23–31)	28 (24–31)	0.012
Strength of identification with:				
‘Gay’ identity ^1^—mean (SD)	1.94 (1.01)	1.78 (1.09)	2.01 (0.96)	0.006
‘Heterosexual’ identity ^1^—mean (SD)	0.75 (0.90)	0.97 (1.00)	0.64 (0.84)	<0.001
Gender identity				<0.001
Male	653 (92.1)	206 (86.2)	447 (95.1)	
Waria, trans woman, or female	56 (7.9)	33 (13.8)	23 (4.9)	
Education				0.047
Junior high school or less	91 (12.9)	25 (10.5)	66 (14.1)	
Senior high school	430 (60.7)	138 (57.7)	292 (62.3)	
University or diploma	187 (26.4)	76 (31.8)	111 (23.7)	
Not reported	1	0	1	
Employment				<0.001
Working	615 (86.7)	192 (80.3)	423 (90.0)	
Studying	28 (4.0)	14 (5.9)	14 (3.0)	
Not working	50 (7.1)	19 (8.0)	31 (6.6)	
Other	16 (2.3)	14 (5.9)	2 (0.4)	
Income per month				0.011
<2 million IDR	204 (29.2)	86 (36.3)	118 (25.5)	
2–3 million IDR	217 (31.0)	74 (31.2)	143 (31.0)	
3–5 million IDR	215 (30.8)	58 (24.5)	157 (34.0)	
5+ million IDR	63 (9.0)	19 (8.0)	44 (9.5)	
Not reported	10	2	8	
Sex work in previous 6 months				0.163
No sex work	510 (71.9)	162 (67.8)	348 (74.0)	
Incidental sex work	120 (16.9)	44 (18.4)	76 (16.2)	
Sex work as main source of income	79 (11.1)	33 (13.8)	46 (9.8)	
Recruited to the study by an outreach worker	650 (91.7)	223 (93.3)	427 (90.9)	0.091
Contact with HIV outreach workers				
Face-to-face (previous 12 months)	634 (89.4)	212 (88.7)	422 (89.8)	0.657
Online (previous 6 months)	551 (77.7)	186 (77.8)	365 (77.7)	0.960
HIV testing				0.001
Never tested	100 (14.1)	49 (20.5)	51 (10.9)	
Tested in previous 12 months	507 (71.5)	154 (64.4)	353 (75.1)	
Tested more than 12 months ago	102 (14.4)	36 (15.1)	66 (14.0)	
HIV status at last HIV test				0.008
HIV-positive	106 (15.0)	32 (13.4)	74 (15.7)	
HIV-negative	391 (55.1)	125 (52.3)	266 (56.6)	
Untested or did not collect result	106 (15.0)	51 (21.3)	55 (11.7)	
Not reported	106 (15.0)	31 (13.0)	75 (16.0)	
Sexual partners in the previous 6 months				
Any men	613 (86.5)	199 (83.3)	414 (88.1)	0.076
Any waria	67 (9.5)	41 (17.2)	26 (5.5)	<0.001
Any women	142 (20.0)	74 (31.0)	68 (14.5)	<0.001
Met male/waria sex partners in the previous 6 months				
Gay mobile applications	414 (58.4)	125 (52.3)	289 (61.5)	0.019
Social media	401 (56.6)	149 (62.3)	252 (53.6)	0.027
Gay bar/club	225 (31.7)	60 (25.1)	165 (35.1)	0.007
Other bar/club	175 (24.7)	56 (23.4)	119 (25.3)	0.581
Gay sauna	143 (20.2)	32 (13.4)	111 (23.6)	0.001
Public area	188 (26.5)	79 (33.1)	109 (23.2)	0.005
Any use of alcohol in previous 6 months	297 (41.9)	89 (37.2)	208 (44.3)	0.073
Any recreational drug use in previous 6 months ^2^	103 (14.5)	39 (16.3)	64 (13.6)	0.335
Any drug use for the purposes of sex in previous 6 months	65 (9.2)	25 (10.5)	40 (8.5)	0.395
PrEP use in HIV-negative respondents ^3^	10 (2.6)	4 (3.2)	6 (2.3)	0.581
Antiretroviral therapy (ART) in HIV-positive respondents ^4^	99 (93.4)	31 (96.9)	68 (91.9)	0.603

^1^ Restricted to cisgender MSM. ^2^ Recreational drug use includes ecstasy, cannabis, crystal methamphetamine, cocaine, or heroin. ^3^ Restricted to HIV-negative respondents. ^4^ Restricted to HIV-positive respondents. NA, non-applicable; IQR, interquartile range; SD, standard deviation; IDR, Indonesian Rupiah; PrEP, pre-exposure prophylaxis.

**Table 2:** Comparison of classifications of the most recent anal intercourse event: Total (*n* = 703); by partner type (romantic partner, *n* = 397; casual partner, *n* = 306); and by respondent place of birth (Bali-born, *n* = 263; non-Bali-born, *n* = 440). ART, antiretroviral therapy; PrEP, pre-exposure prophylaxis.

	Total (*n* = 703)	Romantic Partner (*n* = 397)	Casual Partner (*n* = 306)	*p*-Value for Partner Type Comparison	Bali-Born Respondent (*n* = 263)	Non-Bali-Born Respondent (*n* = 440)	*p*-Value for Place of Birth Comparison
Condom use	397 (56.5)	202 (50.9)	195 (63.7)	0.001	122 (46.4)	275 (62.5)	<0.001
Condomless anal intercourse protected by ART or PrEP	51 (7.3)	33 (8.3)	18 (5.9)	0.873	17 (6.5)	34 (7.7)	0.045
ART in either partner	33 (4.7)	19 (4.8)	14 (4.6)	0.437	11 (4.2)	22 (5.0)	0.120
PrEP in either partner	18 (2.6)	14 (3.5)	4 (1.3)	0.201	6 (2.3)	12 (2.7)	0.263
Condomless anal intercourse unprotected by ART or PrEP	255 (36.3)	162 (40.8)	93 (30.4)	0.004	124 (47.2)	131 (29.8)	<0.001
Any potential risk reduction	177 (25.2)	119 (30.0)	58 (19.0)	0.064	81 (30.8)	96 (21.8)	0.168
Strategic positioning	40 (5.7)	20 (5.0)	20 (6.5)	0.053	23 (8.7)	17 (3.9)	0.221
Concordant HIV status	99 (14.1)	73 (18.4)	26 (8.5)	0.007	52 (19.8)	47 (10.7)	0.769
Withdrawal before ejaculation	86 (12.2)	60 (15.1)	26 (8.5)	0.140	53 (20.2)	33 (7.5)	0.019
No potential risk reduction	78 (11.1)	43 (10.8)	35 (11.4)	0.800	43 (16.3)	35 (8.0)	0.001

**Table 3:** Associations with CLAI unprotected by condoms or biomedical HIV prevention (‘unprotected CLAI events’) using generalised estimating equations (GEE).

	Protected Events (*n* = 448)	Unprotected CLAI Events (*n* = 255)	OR (95% CI)	*p*-Value	aOR (95%CI)	*p*-Value
Participant characteristics						
Age—mean (SD)	28.4 (6.3)	28.0 (6.8)	1.00 (0.97–1.03)	0.964		
Waria or transgender	44 (9.8)	25 (9.8)	0.99 (0.54–1.83)	0.972		
Born in Bali	139 (31.0)	124 (48.6)	1.89 (1.32–2.70)	**<0.001**	2.04 (1.29–3.23)	**0.002**
Employed/working	394 (88.0)	211 (82.8)	1.18 (0.90–1.57)	0.235		
Education: University or diploma	130 (29.0)	59 (23.1)	0.54 (0.30–0.97)	**0.039**	0.40 (0.20–0.83)	**0.013**
Income per month is <2 million IDR	107 (23.9)	90 (35.3)	1.63 (1.12–2.39)	**0.011**	1.21 (0.76–1.92)	0.428
Gay/waria social engagement scale—mean (SD)	3.91 (1.49)	3.54 (1.49)	0.87 (0.78–0.98)	**0.023**	0.95 (0.84–1.11)	0.611
Sex work in previous 6 months	160 (35.7)	116 (45.5)	1.47 (1.02–2.11)	**0.036**	1.53 (0.99–2.35)	0.055
Sex with women and/or waria in previous 6 months	97 (21.7)	91 (35.7)	1.68 (1.14–2.46)	**0.008**	1.01 (0.63–1.62)	0.966
Any recreational drug use in previous 6 months ^1^	70 (15.6)	40 (15.7)	1.01 (0.62–1.64)	0.954		
Used drugs for the purposes of sex in previous 6 months	51 (11.4)	33 (12.9)	1.23 (0.72–2.12)	0.449		
Infrequent contact with HIV outreach workers	205 (45.8)	148 (58.0)	1.60 (1.12–2.28)	**0.009**	1.29 (0.86–1.94)	0.223
Likes using condoms—mean (SD)	3.09 (1.09)	2.45 (1.15)	0.61 (0.52–0.71)	**<0.001**	0.60 (0.50–0.71)	**<0.001**
Belief that withdrawal is an effective HIV prevention method—mean (SD)	0.87 (1.19)	1.63 (1.21)	1.62 (1.40–1.88)	**<0.001**	1.51 (1.26–1.82)	**<0.001**
Likes using withdrawal—mean (SD)	1.02 (1.30)	1.67 (1.28)	1.47 (1.28–1.68)	**<0.001**	1.23 (1.03–1.48)	**0.021**
Partner or event characteristics						
Age—mean (SD)	28.8 (8.6)	29.6 (9.2)	1.01 (0.99–1.03)	0.211		
Waria or transgender	27 (6.0)	19 (7.5)	1.07 (0.55–2.08)	0.840		
Born in Bali	143 (31.9)	111 (43.5)	1.37 (1.00–1.88)	0.053	1.00 (0.66–1.52)	0.990
Romantic partner	235 (52.5)	162 (63.5)	1.53 (1.23–1.90)	**<0.001**	1.77 (1.31–2.39)	**<0.001**
Group sex event	25 (5.6)	25 (9.8)	1.47 (0.84–2.58)	0.177		

^1^ Recreational drug use includes ecstasy, cannabis, crystal methamphetamine, cocaine, or heroin. CLAI, condomless anal intercourse with casual partners; OR, odds ratio; CI, confidence interval; aOR, adjusted odds ratio; SD, standard deviation; IDR, Indonesian Rupiah.
